# Congenital Factor X-Riyadh (Stuart-Prower) Deficiency With Isolated Prothrombin Time Prolongation: A Case Report

**DOI:** 10.7759/cureus.37488

**Published:** 2023-04-12

**Authors:** Badriah G Alasmari, Salma E Tahaelbashir, Mohammed Alomari, Ashwaq M Hommadi, Abdullah Baothman, Saeed M Al-tala

**Affiliations:** 1 Pediatrics, Armed Forces Hospitals Southern Region (AFHSR), Khamis Mushait, SAU; 2 Pediatrics, King Abdullah International Medical Research Center, Jeddah, SAU

**Keywords:** activated partial thromboplastin time (aptt), factor vii deficiency, prothrombin time, factor x-riyadh, coagulation disorders

## Abstract

Factor X (FX) deficiency is an extremely rare autosomal recessive inherited coagulation defect. We report a case of congenital Factor X-Riyadh deficiency discovered during a routine workup before a dental procedure. During routine work-up for dental surgery, prothrombin time (PT) and the international normalized ratio (INR) were prolonged. The prothrombin time (PT) was found to be 78.4 (normal 11-14 seconds) with an international normalized ratio (INR) of 7.83; the activated partial thromboplastin time (APTT) was 30.7 (normal 25-42 seconds). Specific coagulation factor assays confirmed an FX deficiency (<10 % of normal activity) and a mild factor VII deficiency 37% (normal 48%-124%). Molecular genetic analysis of the whole exome sequence (WES) confirmed the diagnosis of FX deficiency (homozygous pathogenic variant c. 271G>A p {Glu91Lys} chr13:113793685). The patient is currently on regular follow-up and is advised to take oral antifibrinolytic medications for any superficial or mucosal bleeding.

## Introduction

The first cases of Factor X deficiency were discovered by Telfer et al. and Hougie in 1956 and 1957, respectively [1,2]. Formerly known as the Stuart-Prower factor, Factor X is a vitamin K-dependent factor. It is a critical enzyme in thrombus formation. A deficiency of Factor X can be an inherited or acquired bleeding disorder. It is an extremely rare inherited coagulation factor disorder, with an estimated one in one million incidences in the general population [1]. However, since it is carried out in an autosomal recessive manner, the hereditary form of FX deficiency may have an impact on more than a hundred genes. A classification system for Factor X deficiency has been proposed based on plasma activity (severe, <10%; moderate, 10% to 40%; mild, >40% of normal activity) [2,3].

When a newborn has a circumcision, umbilical stump bleeding, cerebral hemorrhage, or gastrointestinal bleeding, severe FX deficiency may be present. Inheritable Factor X deficiency is autosomal recessive, and although homozygous individuals may present with menorrhagia, recurrent hematuria, and soft tissue hemorrhage depending on the factor level, heterozygotes often go unnoticed or have only little bleeding following surgery or trauma. Only a few individuals with certain homozygous gene mutations have reported experiencing intracranial bleeding [4]. 

Here we report a case of congenital Factor X deficiency discovered incidentally during a routine coagulation workup before a dental procedure.

## Case presentation

**Figure 1:**
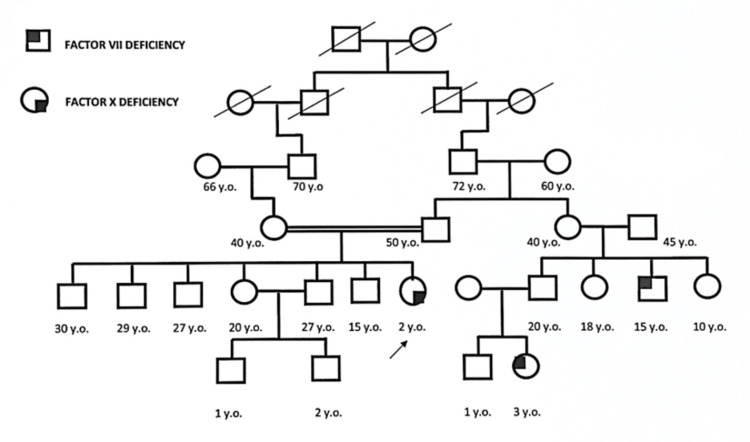
Family pedigree

A two-year-old female with no past medical history was admitted for dental caries operative treatment under general anesthesia. Routine pre-surgery workup, including a complete blood count and bleeding time (BT), were normal. However, the prothrombin time (PT) and INR were prolonged. The PT was found to be 78.4 (normal 11-14 seconds) with an INR of 6.75; the activated partial thromboplastin time (APTT) was 30.7 (normal 25-42 seconds) (Table 1). The patient had no history of bleeding, easy bruising, or joint swelling. There is no history of bleeding following tooth eruption, hematoma post-vaccination, another skin/internal bleeding, or report of umbilical stump bleeding after birth. She was a product of a consanguineous marriage. There was a family history of two cousins having mild Factor VII deficiency, as shown in the pedigree (Figure 1). 

**Table 1:** Coagulation profile *Correction with normal pooled plasma. PT: prothrombin time, APTT: activated partial thromboplastin time, INR: international normalized ratio

Coagulation profile	Reference values (seconds)	Patient's lab values (seconds)
PT (sec)	13	78.4
APTT (sec)	34	30.7
INR	1.2	6.75
Mixing studies*		
PT (sec)		12.7
APTT (sec)		35.7

The rest of the examination was unremarkable. Specific coagulation factor assays confirmed a Factor X deficiency (<10 % of normal activity), representing severe deficiency, and a mild Factor VII deficiency 37% (normal 48%-124%) (Table 2). Molecular genetic analysis of the whole exome sequence (WES) confirmed the diagnosis of Factor X deficiency (homozygous pathogenic variant c. 271G>A p {Glu91Lys} chr13:113793685). Analysis of parental WES data revealed that both parents were heterozygous carriers of this detected FX variant. Parents were investigated for coagulation factor deficiency, and they were also found to have mild Factor VII deficiency. The patient is now on regular follow-up and has been advised to take oral antifibrinolytic medications for any superficial or mucosal bleeding, and in the case of severe bleeding, should be evaluated and advised for FFP to keep FX levels between 10% and 40%.

**Table 2:** Result of coagulation factor assays Interpretation: Severe Factor X deficiency and mild factor VII deficiency

Coagulation Factor	Value (Normal range)
Fibrinogen (mg/dL)	313 mg/dL (160-340)
Factor II (Prothrombin)* (%)	94% (49%-130%)
Factor V* (%)	69% (73%-188%)
Factor VII* (%)	37% (48%-124%)
Factor IX* (%)	78% (53%-129%)
Factor X* (%)	<10% (60%-153%)
Factor XIII*(%)	95% (49%-137%)

## Discussion

Factor X is a glycoprotein that depends on vitamin K. Factor Xa, an active serine protease, is created when it is cleaved. Factor X, a crucial component of the common route that eventually leads to the formation of a clot, is activated by both intrinsic and extrinsic coagulation factors. Tissue thromboplastin, factor VIIa, and calcium work together to activate the extrinsic route. Factor Xa activation will start the process of converting prothrombin to the active form of thrombin, which activates fibrinogen to create fibrin, the primary component of clots. Additionally, activating factors V, VII, and VIII via a positive feedback loop is Factor X. Thus, the process of clot production is ongoing and continues until the bleeding has stopped. Factor VIII and tissue factor may both be rendered inactive by Factor Xa. Antithrombin must first engage in a complicated interaction with Factor Xa before being cleared by the liver [2]. 

Factor X deficiency is also seen in patients with fulminant infections, protein-losing diseases such as burn injury and proteinuria, malignancies (e.g., atypical chronic lymphocytic leukemia [CLL]), and systemic light-chain amyloidosis that occurs in 8.7% to 14% of primary amyloidosis. Most acquired Factor X deficiencies resolve after treatment of the underlying conditions [2,5]. 

The inheritance of Factor X deficiency is an autosomal recessive mode: The gene responsible is positioned in the long arm of chromosome 13. There can be coincidental differences, or there can be a large combined gene loci chromosome 13q34 deletion upon the inheritance of both Factors X and VII [4]. 

Our patient’s factor had functional assays that revealed a mild deficiency of Factor VII (37% of activity). Distinct forms of Factor X deficiency have been identified [6]. A complete absence of FX is incompatible with life. More than 100 Factor X gene mutations have been recognized, most having missense mutations that result in low levels of Factor X activity [7,8]. Our index case has a homozygous pathogenic variant of missense mutations already described in a Saudi family with “Factor X-Riyadh” deficiency [9].

Clinical manifestations correlate well with Factor X levels being <10% of normal in severe disease; these patients tend to have early-life severe bleeding [8].

Investigations for congenital Factor X deficiency include prolongation of coagulation time, thrombin time (TT), PT, and APTT. The kind and severity of the FX level may be determined using a modified PT or APTT test using Factor X-deficient plasma. The PT or APTT may be prolonged by certain Factor X variations. Only prolonged PT, high INR, and normal APTT were seen in the coagulation data. This suggests a lack of Factor X variant type II dysfunctional factors. Bleeding time were normal, and Factor X testing provided supportive evidence. The degree of Factor X activity is largely correlated with the risk of bleeding [10]. Moreover, due to the high consanguinity marriage rate, the prevalence of Factor X deficiency in Saudi Arabia is higher (1.8%) than in other countries [11]. 

In recent years, a four-factor prothrombin complex concentrate (4F-PCC) has been used to restore clotting capabilities, particularly in cases of acquired FX deficit brought on by vitamin K antagonists [12]. Vitamin K administration is not usually effective in congenital factor X deficiency but may be beneficial in acquired cases. In patients with mild disease, the deficiency could benefit from antifibrinolytics or the application of fibrin glue in bleeding sites. There are no specific hemostatic management guidelines for invasive procedures. However, attempts are encouraged to maintain the level at 10%-40% of normal. These were considered adequate for hemostasis before any surgical procedure. The risk of thromboembolic events is higher when Factor X levels exceed 50% of normal. Activities involving severe physical effort should be limited. Liver transplantation has been described as the rare occasion when a child with severe FX deficiency presents with life-threatening bleeding [13]. This case report emphasizes the importance of pre-surgery coagulation workup, including factor functional assays.

## Conclusions

It can be concluded that Factor X (FX) deficiency is an extremely rare autosomal recessive inherited coagulation defect. It can be present without the usual symptoms of bleeding, joint swelling, and skin bruising. In this case, the bleeding time is also normal, but the prothrombin time is only increased. Family history is the major marker of diagnosis due to heredity, but it is confirmed by molecular genetic analysis. The significance of the pre-surgery coagulation workup, which includes a factor functional test, is also highlighted in this case report.
